# Association between single and multiple cardiometabolic diseases and depression: A cross-sectional study of 391,083 participants from the UK biobank

**DOI:** 10.3389/fpubh.2022.904876

**Published:** 2022-08-04

**Authors:** Li Gong, Tianqi Ma, Lingfang He, Guoqiang Lin, Guogang Zhang, Xunjie Cheng, Fanyan Luo, Yongping Bai

**Affiliations:** ^1^Department of Cardiovascular Surgery, Xiangya Hospital, Central South University, Changsha, China; ^2^National Clinical Research Center for Geriatric Disorders, Xiangya Hospital, Central South University, Changsha, China; ^3^Department of Geriatric Medicine, Center of Coronary Circulation, Xiangya Hospital, Central South University, Changsha, China; ^4^Department of Cardiovascular Medicine, Xiangya Hospital, Central South University, Changsha, China; ^5^Department of Cardiovascular Medicine, The Third Xiangya Hospital, Central South University, Changsha, China

**Keywords:** cardiometabolic diseases, depression, accumulative effect, multimorbidity, UK biobank

## Abstract

**Background:**

Individual cardiometabolic diseases (CMDs) are associated with an increased risk of depression, but it's unclear whether having more than one CMD is associated with accumulative effects on depression. We aimed to assess the associations between CMDs and depression and determine the accumulative extent.

**Methods:**

In this cross-sectional study based on UK Biobank, participants with available information on CMDs and depression were enrolled. The history of CMDs was derived from self-reported medical history and electrical health-related records. Depression status was assessed by the aggregation of self-reported history and antidepressant use, depression (Smith), and hospital inpatient diagnoses. Logistic regression models were fitted to assess the association between the number or specific patterns of CMDs and depression and to test the accumulative effect of CMD number, adjusting for confounding factors.

**Results:**

391,083 participants were enrolled in our analyses. After multivariable adjustments, CMDs of different number or patterns were associated with a higher risk of depression compared with the reference group (all *P* < 0.001). In the full-adjusted model, participants with one [odds ratio (OR) 1.26, 95% confidence interval (CI) 1.23–1.29], two (OR 1.50, 95% CI 1.44–1.56), and three or more (OR 2.13, 95% CI 1.97–2.30) CMD(s) had an increased risk of depression. A significant, accumulative dose-related relationship between the number of CMDs and depression was observed (OR 1.25, 95% CI 1.24–1.27). The dose-dependent accumulative relationship was consistent in stratified analyses and sensitivity analyses.

**Conclusions:**

CMDs were associated with a higher risk of depression, and there was an accumulative relationship between CMD number and depression.

## Introduction

Depressive disorders are a main and increasing cause of disability, affecting about 280 million people worldwide (1). Besides the high prevalence, they also have a negative influence on social and professional functioning, physical health, and quality of life, and often co-exist with cardiometabolic diseases (CMDs) (2, 3).

There are numerous studies suggesting associations between CMDs and depression. Individual CMDs like diabetes, hypertension, coronary artery disease (CAD), and stroke have been demonstrated to relate to depression (2–4). CMDs and depression may share common environmental factors and common risk factors such as inflammation and metabolic factors (5, 6), or the mechanism underlying this comorbidity is simply shared genetic variants and pathways (7). However, studies demonstrated that CMDs play an important role in the development of depression (8–10).

Although there is broad agreement that individual CMDs are related to depression, it is uncertain whether there is a cumulative effect on the risk of depression when an individual suffers from more than one CMD. Considering the increasing prevalence of cardiometabolic multimorbidity globally (11, 12), the purpose of this study is to assess the association of multiple CMDs with depression and to elucidate whether there is a cumulative effect of CMDs on depression in the UK Biobank cohort. This question is of great importance since there is a growing number living with more than one CMDs.

## Methods

### Study population and design

This study utilized data from the UK Biobank. UK Biobank is a large population-based prospective study, recruiting over 500,000 participants aged 40–69 years from 2006 to 2010 across the United Kingdom. Providing informed consent, the participants finished questionnaires, interviews, physical measurements and offered biological samples to provide extensive information on demographics, health-related lifestyles, medical history, and anthropometry. Health outcomes including disease diagnoses, operations, and deaths were accessed *via* linkages to electrical health-related records. UK Biobank has been approved by the National Health Service (NHS) National Research Ethics Service (approval letter dated 17th June 2011, Ref 11/NW/0382).

Data from 502,415 participants of UK Biobank was available. In this cross-sectional study, we enrolled participants with available information on the status of CMDs and depression disorders and other involved covariates, and we excluded people with other psychosis phenotypes (psychosis and bipolar disorder) at recruitment (*n* = 5,678, definitions shown in Supplementary materials, Section 1). Finally, 391,083 participants were involved in our analyses.

### Assessment of exposure

The interested exposure was the status of CMDs [diabetes, hypertension, stroke, and CAD (including myocardial infarction and stable angina)] at recruitment in this study. The detailed definitions are given in Supplementary Table 1. In brief, self-reported medical history, medication use, operation history, and electrical health-related records were aggregated to derive the status of CMDs and corresponding diagnosed date. Participants were grouped by the number of CMDs at recruitment (0–4), and those with 3 or 4 conditions were merged into ≥3 conditions in later analyses because of the small sample size of 4 diseases (*n* = 283). In further analyses, we also categorized participants exclusively according to specific patterns of CMDs: (a). No CMDs; (b). Diabetes; (c). Hypertension; (d). CAD; (e). Stroke; (f). Hypertension + diabetes; (g). Hypertension + CAD; (h). Hypertension + stroke; (i). Hypertension + diabetes + CAD. For adequate statistical power, only patterns with ≥1,000 cases were involved.

### Assessment of outcomes

The primary outcome was depression at recruitment. Here, we adopted definitions from Glanville et al. (13) and used 4 measurements to identify depression [(a). Self-reported history of depression; (b). Antidepressant using history; (c). Depression defined by Smith et al. (14); (d). Diagnosis of hospital inpatient coded by the International Classification of Disease version 10 (ICD-10)]. Participants who met ≥1 criteria were considered with depression. Detailed definitions, field IDs, and codes are provided in Supplementary materials, Section 1.

We adopted 3 depression phenotypes defined by Smith et al. (14) as secondary outcomes. At baseline, 172,751 participants finished an extended touchscreen questionnaire about psychosocial questions. According to the severity and frequency of depressive symptoms (Supplementary materials, Section 2), these participants were classified to have: (a). Probable lifetime (single) episode of major depression; (b). Probable recurrent major depressive disorder (moderate); (c). Probable recurrent major depressive disorder (severe).

### Other covariates

Age, ethnicity, sex, education degree, Townsend deprivation index, and lifestyles were derived from population characteristics and touchscreen questionnaires. Education was dichotomized into University/college/other professional qualifications degrees and others depending on self-reported highest qualification achieved. Townsend deprivation index was derived from the postcode of residence, proving a measure of material deprivation. Body mass index (BMI, kg/m^2^) was calculated as measured body weight (kg) divided by square of height (m^2^). Alcohol consumption frequency was dichotomized into ≤2 and >2 times per week. Diet was assessed by a healthy diet score adopted from the American Heart Association Guidelines (15), involving the consumption of fruit and vegetable, fish, processed, and red meat (presented in Supplementary Table 2).

### Statistical analyses

The population characteristics were described according to the number and patterns of CMDs, respectively. Continuous variables were expressed as mean [standard deviation (SD)], and categorical ones were expressed as number (percentage, %).

First, a series of subset data were extracted and logistic regression models were fitted to assess the association between the number or specific patterns of CMDs and risk of depression, setting participants without CMDs as the reference group. Then, to assess the accumulative effects of disease number, a continuous variable was generated, with value 0 for no disease, 1 for single disease, 2 for 2 diseases, and 3 for ≥3 conditions, and this variable was used to fit logistic regression models of the whole crowd. Each model had 3 settings: a base model adjusted for age, sex, and ethnicity; a partially-adjusted model further adjusted for Townsend deprivation index and education degree; a fully-adjusted model further adjusted for healthy diet, current smoking status, alcohol consumption, and BMI. In later analyses, we extracted subsets of participants with information on secondary outcomes and re-fitted the models above.

To investigate modification effects of other confounding factors on the accumulative association of CMDs number with depression, stratified analyses were conducted according to sex (male or female), age (<60 or ≥60 years), ethnicity (white or others), education (college and higher or others), Townsend deprivation index ( ≤0 or >0), BMI (<30 or ≥30), current smoking (yes or no), alcohol consumption frequency ( ≤2/week or >2/week), and healthy diet score (0 or 1).

Moreover, to test the robustness of the results, we performed several sensitivity analyses. Firstly, because participants who engaged in physical exercise were less likely to develop depression (16), we further adjusted for physical activity in the full-adjusted models. Physical activity was dichotomized according to whether a person met the 2017 UK Physical activity guidelines of 150 min of walking or moderate activity or 75 min of vigorous activity per week (17). Secondly, we broadened the definition of depression by adding a measurement of help-seeking. Participants who had ever seen a psychiatrist for nerves, anxiety, tension, or depression were thought to fulfill this criterion. Thirdly, we widened the definition of depression phenotypes by including the measurement of two-item Patient Health Questionnaire (PHQ-2) (18). At recruitment, participants were asked the frequency of depressed mood and unenthusiasm/disinterest in last 2 weeks. For the two questions, they could select the following answers: “Prefer not to answer/Do not know” (considered having miss information), “Not at all” (scored 0), “Several days” (scored 1), “More than half the days” (scored 2), or “Nearly every day” (scored 3). Participants who had a combined score of ≥3 were considered with probable current depression. Fourthly, as participants with different levels of education may pay attention to health variously, we assessed education as a more fine-grained ordinal variable: college/University/other professional qualification; A-levels/AS-levels; O-levels/GCSEs; CSEs; NVQ/HND/HNC.

We performed all analyses using R software version 4.1.0. The results derived from logistic regression models are presented as odds ratio (OR) and 95% confidence interval (CI). All *P* values in our analyses were two-sided, and the difference was considered statistically significant when *P* values < 0.05.

## Results

### Baseline patients' characteristics

Finally, 391,083 participants were involved in our study, with a mean age of 56.16 years (SD = 8.07). Among them, 176,893 (45.2%) were male, 371,252 (94.9%) were white, and 225,407 (57.6%) had a college-equal degree. 273,803 (70.0%) participants were free of CMDs, and 91,254 (23.3%), 22,107 (5.7%), and 3,919 (1.0%) suffered from 1, 2, and ≥3 conditions, respectively. In terms of depression, 59,182 (15.1%) participants met our definition and 25,098 (6.4%) used antidepressant. The distribution of population characteristics across number of CMDs is shown in Table 1. Overall, along with the increasing number of conditions, patients were more likely to be older, male, non-white, more deprived, current-smoking, have a lower education degree, higher BMI, and lower alcohol consumption. The proportion of depression and antidepressant use also increased according to disease number. The descriptive statistics according to specific patterns of CMDs are presented in Supplementary Table 3, with a similar tendency observed.

**Table 1:** Population characteristics according to number of CMDs.

**Characteristics**	**0**	**1**	**2**	≥3*
Participants, *n* (%)	273,803 (70.0%)	91,254 (23.3%)	22,107 (5.7%)	3,919 (1.0%)
Age (years), mean (SD)	54.84 (8.03)	58.70 (7.38)	60.88 (6.65)	62.17 (6.05)
Male, *n* (%)	114,768 (41.9)	44,861 (49.2)	14,389 (65.1)	2,875 (73.4)
White ethnicity, *n* (%)	261,198 (95.4)	86,212 (94.5)	20,369 (92.1)	3,473 (88.6)
Townsend deprivation index, mean (SD)	−1.63 (2.89)	−1.52 (2.98)	−1.16 (3.16)	−0.57 (3.40)
College and higher degree #, *n* (%)	161,285 (58.9)	50,450 (55.3)	11,728 (53.1)	1,944 (49.6)
Healthy diet, *n* (%)	153,836 (56.2)	52,632 (57.7)	12,696 (57.4)	2,208 (56.3)
Current smoking, *n* (%)	26,193 (9.6)	7,819 (8.6)	2,084 (9.4)	441 (11.3)
Alcohol consumption frequency (times/week) ≥3, *n* (%)	127,628 (46.6)	43,119 (47.3)	9,156 (41.4)	1,325 (33.8)
Body mass index (kg/m^2^), mean (SD)	26.38 (4.26)	28.68 (5.01)	30.22 (5.56)	31.29 (5.66)
Depression disorder, *n* (%)	38,705 (14.1)	15,436 (16.9)	4,097 (18.5)	944 (24.1)
Antidepressant use, *n* (%)	15,212 (5.6)	7,172 (7.9)	2,177 (9.8)	537 (13.7)

CMDs means cardiometabolic diseases.

^*^CMD number ≥3: since the small sample size of patients with concurrent 4 CMDs (n = 283), conditions of 3 and 4 CMDs were analyzed in combination as CMD number ≥3.

#College and higher degree included college or university degree and other professional qualifications such as nursing and teaching.

### Association between CMDs and depression

The association between CMD number and the risk of depression is shown in Table 2. In the base model, CMDs were associated with a higher risk of depression (all *P* < 0.001), and a significant, accumulative dose-related relationship between the number of CMDs and depression was observed (OR 1.36, 95% CI 1.34–1.38, *P* < 0.001). The associations remained significant after adjustments for other covariates. In full-adjusted models, the ORs for depression increased from 1.26 of 1 disease to 2.13 of ≥3 diseases, and the accumulative effect of CMD number was attenuated but remained significant (OR 1.25, 95% CI 1.24–1.27, *P* < 0.001).

**Table 2:** The association between CMD number and depression §.

**CMD numbers**	**Base model** *			**Partially-adjusted model #**			**Full-adjusted model &**		
	**OR**	**95% CI**	***P***	**OR**	**95% CI**	***P***	**OR**	**95% CI**	***P***
1	1.37	(1.34; 1.39)	<0.001	1.35	(1.32; 1.38)	<0.001	1.26	(1.23; 1.29)	<0.001
2	1.76	(1.70; 1.83)	<0.001	1.70	(1.64; 1.77)	<0.001	1.50	(1.44; 1.56)	<0.001
≥3	2.66	(2.46; 2.87)	<0.001	2.50	(2.32; 2.70)	<0.001	2.13	(1.97; 2.30)	<0.001
Accumulative dose effect	1.36	(1.34; 1.38)	<0.001	1.34	(1.32; 1.36)	<0.001	1.25	(1.24; 1.27)	<0.001

CMD means cardiometabolic diseases; OR means odds ratio; 95% CI means 95% confidence interval.

§Reference group: participants free of CMDs (except for the accumulative dose effects of CMD number).

^*^Base model: Adjusted for age, sex, and ethnicity.

#Partially-adjusted model: Further adjusted for Townsend deprivation score, and education degree.

&Full-adjusted model: Further adjusted for healthy diet, current smoking status, alcohol consumption, and body mass index.

We also analyzed the associations between CMD patterns and depression (seen in Supplementary Table 4). Compared with the reference group, participants with CMDs of different patterns had a higher risk of depression, and patterns with more CMDs were associated with increased risks of depression. For example, The ORs increased from 1.36 (CAD only) to 1.51 (CAD + hypertension) to 1.95 (CAD + hypertension + diabetes) in the full-adjusted model.

### Association between CMDs and probable lifetime depression

The associations between CMD number and the risk of three probable lifetime depressive disorders defined by Smith et al. (14) are presented in Table 3. Except for the association between 2 CMDs and single episode of probable major depression, the existence of CMDs was significantly associated with a higher risk for each secondary outcome, irrespective of the adjustments for covariates in models of three settings. The dose-dependent accumulative relationship was significant for all probable lifetime depression, and seemed to be stronger for recurrent and severe depressive disorder [full-adjusted OR 1.10, 95% CI 1.06–1.14, *P* < 0.001 for single episode of major depression, and full-adjusted OR 1.21, 95% CI 1.16–1.25, *P* < 0.001 for the recurrent major depressive disorder (severe)].

**Table 3:** The association between CMD number and probable lifetime depression §.

**Secondary outcomes**	**CMD number**	**Base model** *			**Partially-adjusted model #**			**Full-adjusted model &**		
		**OR**	**95% CI**	***P***	**OR**	**95% CI**	***P***	**OR**	**95% CI**	***P***
Single episode ¶	1	1.18	(1.12; 1.25)	<0.001	1.18	(1.12; 1.25)	<0.001	1.14	(1.08; 1.20)	<0.001
	2	1.19	(1.08; 1.32)	0.001	1.18	(1.07; 1.31)	0.001	1.09	(0.98; 1.21)	0.099
	≥3	1.54	(1.24; 1.91)	<0.001	1.51	(1.22; 1.87)	<0.001	1.36	(1.09; 1.69)	0.006
	Accumulative dose effect	1.14	(1.10; 1.18)	<0.001	1.14	(1.10; 1.18)	<0.001	1.10	(1.06; 1.14)	<0.001
Recurrent moderate ⋇	1	1.23	(1.18; 1.29)	<0.001	1.22	(1.17; 1.28)	<0.001	1.15	(1.10; 1.20)	<0.001
	2	1.38	(1.27; 1.50)	<0.001	1.35	(1.24; 1.46)	<0.001	1.22	(1.12; 1.32)	<0.001
	≥3	1.80	(1.52; 2.13)	<0.001	1.72	(1.46; 2.04)	<0.001	1.50	(1.27; 1.78)	<0.001
	Accumulative dose effect	1.21	(1.17; 1.24)	<0.001	1.20	(1.16; 1.23)	<0.001	1.13	(1.10; 1.17)	<0.001
Recurrent severe $	1	1.33	(1.26; 1.41)	<0.001	1.30	(1.23; 1.38)	<0.001	1.25	(1.18; 1.33)	<0.001
	2	1.51	(1.37; 1.67)	<0.001	1.43	(1.29; 1.58)	<0.001	1.33	(1.20; 1.48)	<0.001
	≥3	2.46	(2.05; 2.96)	<0.001	2.22	(1.84; 2.67)	<0.001	1.99	(1.65; 2.40)	<0.001
	Accumulative dose effect	1.30	(1.25; 1.34)	<0.001	1.26	(1.22; 1.31)	<0.001	1.21	(1.16; 1.25)	<0.001

CMD means cardiometabolic diseases; OR means odds ratio; 95% CI means 95% confidence interval.

§Reference group: participants free of CMDs (except for the accumulative dose effects of CMD number).

^*^Base model: Adjusted for age, sex, and ethnicity.

#Partially-adjusted model: Further adjusted for Townsend deprivation score, and education degree.

&Full-adjusted model: Further adjusted for healthy diet, current smoking status, alcohol consumption, and body mass index.

¶Single episode: Probable lifetime (single) episode of major depression.

⋇Recurrent moderate: Probable recurrent major depressive disorder (moderate).

$Recurrent severe: Probable recurrent major depressive disorder (severe).

Then we analyzed the associations between CMD patterns and secondary outcomes of depression (seen in Supplementary Table 5). Consistent with the results above, the CMD-related risk of secondary outcomes was stronger among patients with concurrent CMDs and for recurrent and severe phenotypes. For patients with concurrent hypertension, diabetes, and CAD, the OR for the recurrent, severe major depressive disorder was 1.89 (95% CI 1.48–2.40, *P* < 0.001).

### Stratified analyses and sensitivity analyses

**Figure 1:**
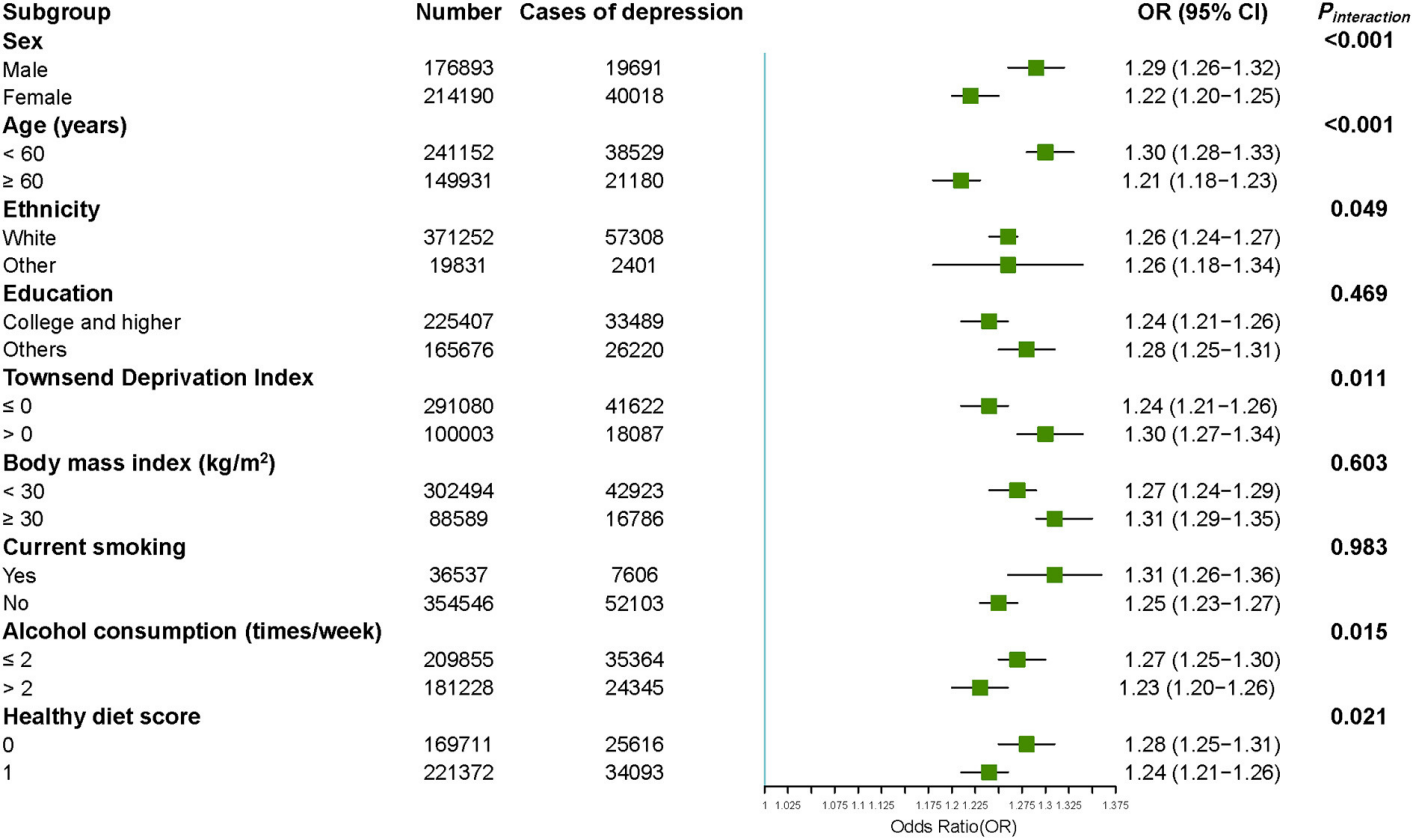
Accumulative association between CMD number and risk of depression stratified by potential risk factors. The ORs for the risk of depression were derived from logistic regression models. Results were adjusted for age, sex, ethnicity, Townsend deprivation index, education degree, healthy diet, current smoking status, alcohol consumption, and body mass index conditionally. CMD means cardiometabolic disease, OR means odd ratio, 95% CI means 95% confidence interval.

As shown in Figure 1, we performed stratified analyses according to potential modifying factors. The accumulative dose effect existed in all subgroups stratified by sex, age, ethnicity, education, Townsend deprivation index, smoking status, alcohol consumption frequency, and healthy diet, and was stronger among participants who were male (*P*_*interaction*_ < 0.001), younger (*P*_*interaction*_ < 0.001), more deprived (*P*_*interaction*_ = 0.011), consumed alcohol ≤2 times/week (*P*_*interaction*_ = 0.015), and had an unadvisable diet (*P*_*interaction*_ = 0.021). No significant differences were found in ethnicity, education, BMI, and current smoking status (all *P*_*interaction*_ > 0.05).

Sensitivity analyses showed no substantial change in the association between depression and CMD number or patterns when we added physical activity as a covariate into the full-adjusted model (seen Supplementary Table 6), widened the definition of depression by adding a measurement of help-seeking or PHQ-2 (seen Supplementary Tables 7, 8), or adjusted education into a more fine-grained ordinal variable (seen Supplementary Table 9).

## Discussion

### Findings and comparison with previous researches

In this cross-sectional study, we found that compared with people free of CMDs, patients with CMDs were at a higher risk of depression disorder, and a dose-dependently accumulative relationship of CMDs with depression existed after adjustments for potential confounders. The accumulative association was stronger for recurrent and severe depression phenotype and were modified by age, sex, Townsend deprivation score, diet, and alcohol consumption.

Our results that patients of CMDs had a higher risk for depression were supported by previous studies. According to researches based on individual CMD, depression was common in survivors of acute myocardial infarction (19), and up to two-thirds of patients develop depression after MI (20), and about one in five adults with type 2 diabetes present depression (21). And researches exploring association of cardiometabolic multimorbidity and depression also demonstrated that cardiometabolic multimorbidity are related to depression (22, 23). However, these researches didn't concentrate on the accumulative effects of concurrent CMDs. Considering the increasing prevalence of cardiometabolic multimorbidity, we assessed the associations of CMD number and specific patterns with the risk of depression and observed significant dose-dependent accumulative effects of CMD number on depression.

In terms of the accumulative effects of cardiometabolic multimorbidity, existing evidence suggested that it was associated with worse functional status, declining cognitive function, and poor prognosis (24, 25). According to The Emerging Risk Factors Collaboration's findings, a history of cardiometabolic multimorbidity was associated with a cumulatively increased risk of all-cause mortality. Compared with those without CMDs, patients with 3 conditions had nearly 7 times higher risk of death (24). Our results extended the detrimental effects of concurrent CMDs to psychological outcomes, shedding light on the necessity of mental intervention for patients with concurrent CMDs. Even though in the current study, we have to admit the heterogenicity across different CMD patterns, the analyses of additive effects based on CMD numbers describe an intuitive phenomenon that the risk of depression increases along with the occurrences of new-onset CMDs, irrespective of original disease conditions, which might be more readily comprehensible for policymakers, clinicians, and patients. And the pattern-specific associations with depression risk provide complementary information.

By now, the Mental Health Questionnaire (MHQ) derivation of lifetime depression is the gold-standard for depression phenotyping (13). However, participants didn't complete the MHQ at the time of baseline assessment, and only a subset of 157 366 participants completed it in 2017 (26). Previous studies of the UK biobank adopted various diagnostic criteria for depression. Some used single criterion such as the study by Khandaker GM et al. (6) using Patient Health Questionnaire (PHQ)-9 (27) and the study by Davidson KW et al. using Patient Health Questionnaire-2 (PHQ-2) (28). Some adopted multiple measures such as the study by McIntosh AM et al. (25) including broad depression phenotype, probable major depressive disorder (MDD) phenotype, and ICD-coded MDD phenotype (29). Here we adopted definitions from Glanville et al. (13) and aggregated information on self-reported history, medication, mental-health questionnaires, and ICD-10 records to identify depression. Due to the different diagnostic criteria for depression, more researches need to be done to confirm our conclusions. Given this, we widened the definition of depression by adding criteria of “help-seeking for a psychiatrist” or PHQ-2 in two sensitivity analyses respectively to test our findings. And we also utilized the criteria defined by Smith et al. (14) and estimated the accumulative association of CMDs with depression phenotypes of different frequency and severity.

There are several possible explanations for the accumulative associations between cardiometabolic multimorbidity and depression risk. First, CMDs share similar and overlapping etiology, risk factors, and metabolic disorders. Patients with CMDs are usually obsessed by the chronic disease course, declined physical function, and dependence on social supports, and they are prone to suffer from negative emotional burdens such as worry, frustration, and burnout (21, 30), which might accumulate along with the coexistence of CMDs (31). Second, multimorbidity and related polypharmacy are associated with poor health outcomes, high readmission rate, and a heavy financial burden (32), which contributes to the psychological stress of patients suffering from cardiometabolic multimorbidity. In addition, the common risk factors shared by CMDs and depression might contribute to the accumulative association between CMD number and depression. According to our findings and previous evidence (33), patients with CMDs are more likely to have unhealthy lifestyles including smoking, excessive alcohol consumption, unhealthy diet, and sedentariness, which are also risk factors of depression (34).

Furthermore, in subgroup analyses stratified by confounding factors, we observed stronger CMD-related dose effects among people who were male, younger, more deprived, who consumed less alcohol, and who had an unadvisable diet. The differences in the association between CMD status and depression disorder among subgroups might be attributed to the features of certain subgroups. For instance, subgroups of female (35) or excessive consumption of alcohol (36), are at an internally higher risk of depression, and the CMD-related effects might be diluted, leading to a relatively stronger association observed for subgroups of male and low alcohol consumption. And for young people or people who had a lower socioeconomic status, suffering from CMDs means lower life quality, poorer prognosis, and heavier economic burden, which could contribute to greater psychological stress and a higher risk of depression. In addition, since the results of subgroup analyses are tentative, it needs to be cautious to interpret the differences in the associations observed among subgroups, and further researches are needed to verify the potential discrepancies in terms of age, sex, socioeconomic status, lifestyle, etc.

The major discovery of this study is the accumulative effects of CMDs count on depression. According to our results, patients with cardiometabolic multimorbidity were more prone to suffer from depressive disorder, especially recurrent and severe symptoms. Our findings highlight the importance of awareness of depression in patients with CMDs, especially those with more than one condition. Considering the worse clinical prognosis of the comorbidity of depression and CMDs (37), integrated biopsychosocial management is necessary when people are diagnosed with CMDs (38). Besides the comorbidity of CMDs, more multimorbidity of chronic physical health disorders is rising as people live longer and access to medical support is easier. It's of high social value to pay attention to accumulative effect for we can take effective measures in advance to prevent and treat diseases.

### Strengths and limitations

Our study has several strengths. Firstly, the large scale of UK Biobank enabled us to conduct analyses according to the number and specific patterns of CMDs based on adequate sample size and to test for potential accumulative effects of coexistence of ≥2 conditions. Secondly, utilizing information in the self-reported medical history of diagnoses, medication, and operation, and inpatient health-related records of diagnoses and operations, our definitions of CMDs were refined, mitigating the misclassification of patients with CMDs. Thirdly, for the assessment of depression disorder, we aggregated 4 measurements including the self-reported history of depression, antidepressant use, inpatient diagnoses coded by ICD-10, and depression defined by Smith et al. (14) derived from questionnaires at recruitment. In further analyses for secondary outcomes, we enrolled 3 ordered phenotypes of depression disorder according to the recurrence and severity of symptoms, and we found that CMD-related increase was stronger for recurrent and severe phenotypes, enriching the dimension of our results.

However, this study has limitations. Firstly, participants of the UK biobank are mostly white, limiting the generalizability of our results to other ethnicities and countries. And evidence suggested that they were not representative of the sampling population, with a “healthy volunteer” selection bias (39). Secondly, as a cross-sectional study, this research cannot establish a causal relationship between CMD and depression, since both causation and reverse causation are plausible. Patients with CMDs may get depressive for the burden of incurable chronic diseases, and people with depression are less likely to engage in healthy lifestyles and ask for medical help that protects against CMDs (40). Thirdly, in our models, all effect sizes attenuated to some extent from the base models after adjustments for factors such as deprivation, education, and lifestyles, indicating the confounding effects of covariates involved. Even though we have performed several sensitivity analyses to test the robustness of our findings, there are residual confounders remained, which might introduce deviations to our results.

## Conclusion

Overall, the existence of CMDs were dose-dependently and accumulatively associated with an increased risk of depression disorder. Our findings extended the deleterious effects of concurrent CMDs to psychological outcomes, and further prospective researches are necessary to prove our results.

## Data availability statement

The data analyzed in this study is subject to the following licenses/restrictions: Data described in the article, code book, and analytic code will be made available upon request pending the permission of UK Biobank. Requests to access these datasets should be directed to https://www.ukbiobank.ac.uk/.

## Ethics statement

The studies involving human participants were reviewed and approved by the National Health Service National Research Ethics Service. The patients/participants provided their written informed consent to participate in this study.

## Author contributions

LG: conceptualization, validation, investigation, data curation, and writing–original draft preparation. TM: methodology, validation, formal analysis, investigation, data curation, writing–original draft preparation, and visualization. LH: methodology, validation, investigation, data curation, and writing–review and editing. GL: software, validation, data curation, and writing–review and editing. GZ: investigation, resources, and writing–review and editing. XC: conceptualization, formal analysis, writing–review and editing, supervision, and funding acquisition. FL: conceptualization, formal analysis, writing–review and editing, and supervision. YB: conceptualization, supervision, project administration, and funding acquisition. All authors gave final approval and agree to be accountable for all aspects of the work ensuring integrity and accuracy.

## Funding

This study was funded by the National Natural Science Foundation of China (YB, Grant Number 81822004), the National Key Research and Development Program of China (YB, Grant Number 2020YFC2008002), the Science and Technology Innovation Program of Hunan Province (YB, Grant Number 2020RC4006, XC, Grant Number 2021RC2014, and LG, Grant Number 2021RC2032), and the Project of Innovation-driven Plan in Central South University (YB, Grant Number 2020CX017). The funders had no role in study design, data collection, and analysis, decision to publish, or preparation of the manuscript.

## Conflict of interest

The authors declare that the research was conducted in the absence of any commercial or financial relationships that could be construed as a potential conflict of interest.

## Publisher's note

All claims expressed in this article are solely those of the authors and do not necessarily represent those of their affiliated organizations, or those of the publisher, the editors and the reviewers. Any product that may be evaluated in this article, or claim that may be made by its manufacturer, is not guaranteed or endorsed by the publisher.

## References

[1] Diseases GBD Injuries C. Global burden of 369 diseases and injuries in 204 countries and territories, 1990–2019: a systematic analysis for the global burden of disease study 2019. Lancet. (2020) 396:1204–22. 10.1016/S0140-6736(20)30925-9 33069326 PMC7567026

[2] Martin DJ Ul-Haq Z Nicholl BI Cullen B Evans J Gill JM. Cardiometabolic disease and features of depression and bipolar disorder: population-based, cross-sectional study. Br J Psychiatry. (2016) 208:343–51. 10.1192/bjp.bp.114.157784 26795427

[3] Chirinos DA Murdock KW LeRoy AS Fagundes C. Depressive symptom profiles, cardio-metabolic risk and inflammation: results from the midus study. Psychoneuroendocrinology. (2017) 82:17–25. 10.1016/j.psyneuen.2017.04.011 28486177 PMC5833295

[4] Scott KM. Depression, anxiety and incident cardiometabolic diseases. Curr Opin Psychiatry. (2014) 27:289–93. 10.1097/YCO.0000000000000067 24840158

[5] Alshehri T Mook-Kanamori DO Willems van Dijk K Dinga R Penninx B Rosendaal FR. Metabolomics dissection of depression heterogeneity and related cardiometabolic risk. Psychol Med. (2021). 10.1017/S0033291721001471. [Epub ahead of print]. 34078486 PMC9874986

[6] Khandaker GM Zuber V Rees JMB Carvalho L Mason AM Foley CN. Shared mechanisms between coronary heart disease and depression: findings from a large Uk general population-based cohort. Mol Psychiatry. (2020) 25:1477–86. 10.1038/s41380-019-0395-3 30886334 PMC7303009

[7] Wong BC Chau CK Ao FK Mo CH Wong SY Wong YH. Differential associations of depression-related phenotypes with cardiometabolic risks: polygenic analyses and exploring shared genetic variants and pathways. Depress Anxiety. (2019) 36:330–44. 10.1002/da.22861 30521077

[8] Robinson RG Jorge RE. Post-stroke depression: a review. Am J Psychiatry. (2016) 173:221–31. 10.1176/appi.ajp.2015.15030363 26684921

[9] Anderson RJ Freedland KE Clouse RE Lustman PJ. The prevalence of comorbid depression in adults with diabetes: a meta-analysis. Diabetes Care. (2001) 24:1069–78. 10.2337/diacare.24.6.1069 11375373

[10] de Jonge P Roy JF Saz P Marcos G Lobo A Investigators Z. Prevalent and incident depression in community-dwelling elderly persons with diabetes mellitus: results from the Zarademp project. Diabetologia. (2006) 49:2627–33. 10.1007/s00125-006-0442-x 17019601

[11] Zhang D Tang X Shen P Si Y Liu X Xu Z. Multimorbidity of cardiometabolic diseases: prevalence and risk for mortality from one million Chinese adults in a longitudinal cohort study. BMJ Open. (2019) 9:e024476. 10.1136/bmjopen-2018-024476 30833320 PMC6443196

[12] Sakakibara BM Obembe AO Eng JJ. The prevalence of cardiometabolic multimorbidity and its association with physical activity, diet, and stress in Canada: evidence from a population-based cross-sectional study. BMC Public Health. (2019) 19:1361. 10.1186/s12889-019-7682-4 31651286 PMC6814029

[13] Glanville KP Coleman JRI Howard DM Pain O Hanscombe KB Jermy B. Multiple measures of depression to enhance validity of major depressive disorder in the UK biobank. BJPsych open. (2021) 7:e44. 10.1192/bjo.2020.145 33541459 PMC8058908

[14] Smith DJ Nicholl BI Cullen B Martin D Ul-Haq Z Evans J. Prevalence and characteristics of probable major depression and bipolar disorder within UK biobank: cross-sectional study of 172,751 participants. PLoS ONE. (2013) 8:e75362. 10.1371/journal.pone.0075362 24282498 PMC3839907

[15] Benjamin EJ Blaha MJ Chiuve SE Cushman M Das SR Deo R. Heart disease and stroke statistics-2017 update: a report from the American heart association. Circulation. (2017) 135:e146–603. 10.1161/CIR.0000000000000491 28122885 PMC5408160

[16] Guck TP Kavan MG Elsasser GN Barone EJ. Assessment and treatment of depression following myocardial infarction. Am Fam Physician. (2001) 64:641–8. 11529263

[17] Cassidy S Chau JY Catt M Bauman A Trenell MI. Cross-sectional study of diet, physical activity, television viewing and sleep duration in 233,110 adults from the UK biobank; the behavioural phenotype of cardiovascular disease and type 2 diabetes. BMJ Open. (2016) 6:e010038. 10.1136/bmjopen-2015-010038 27008686 PMC4800116

[18] Foster M Niedzwiedz CL. Associations between multimorbidity and depression among breast cancer survivors within the UK biobank cohort: a cross-sectional study. BMC Cancer. (2021) 21:650. 10.1186/s12885-021-08409-z 34058985 PMC8167936

[19] Thombs BD Bass EB Ford DE Stewart KJ Tsilidis KK Patel U. Prevalence of depression in survivors of acute myocardial infarction. J Gen Intern Med. (2006) 21:30–8. 10.1111/j.1525-1497.2005.00269.x 16423120 PMC1484630

[20] Ziegelstein RC. Depression in patients recovering from a myocardial infarction. Jama. (2001) 286:1621–7. 10.1001/jama.286.13.1621 11585486

[21] Owens-Gary MD Zhang X Jawanda S Bullard KM Allweiss P Smith BD. The importance of addressing depression and diabetes distress in adults with type 2 diabetes. J Gen Intern Med. (2019) 34:320–4. 10.1007/s11606-018-4705-2 30350030 PMC6374277

[22] Li C Peng W Li M Li X Yang T Yan H. Exploring the relationship between depression and different multimorbidity patterns among older people covered by long-term care insurance in Shanghai, China. Psychogeriatrics. (2022) 22:99–107. 10.1111/psyg.12783 34743400 PMC9297888

[23] Dibato JE Montvida O Zaccardi F Sargeant JA Davies MJ Khunti K. Association of cardiometabolic multimorbidity and depression with cardiovascular events in early-onset adult type 2 diabetes: a multiethnic study in the US. Diabetes care. (2021) 44:231–9. 10.2337/dc20-2045 33177170

[24] Emerging Risk Factors C Di Angelantonio E Kaptoge S Wormser D Willeit P Butterworth AS. Association of cardiometabolic multimorbidity with mortality. JAMA. (2015) 314:52–60. 10.1001/jama.2015.7008 26151266 PMC4664176

[25] Lyall DM Celis-Morales CA Anderson J Gill JM Mackay DF McIntosh AM. Associations between single and multiple cardiometabolic diseases and cognitive abilities in 474 129 UK biobank participants. Eur Heart J. (2017) 38:577–83. 10.1093/eurheartj/ehw528 28363219 PMC5381595

[26] Davis KAS Coleman JRI Adams M Allen N Breen G Cullen B. Mental health in UK biobank - development, implementation and results from an online questionnaire completed by 157 366 participants: a reanalysis. BJPsych open. (2020) 6:e18. 10.1192/bjo.2019.100 32026800 PMC7176892

[27] Milaneschi Y Kappelmann N Ye Z Lamers F Moser S Jones PB. Association of inflammation with depression and anxiety: evidence for symptom-specificity and potential causality from UK biobank and nesda cohorts. Mol Psychiatry. (2021) 26:7393–402. 10.1038/s41380-021-01188-w 34135474 PMC8873022

[28] Harshfield EL Pennells L Schwartz JE Willeit P Kaptoge S Bell S. Association between depressive symptoms and incident cardiovascular diseases. JAMA. (2020) 324:2396–405. 10.1001/jama.2020.23068 33320224 PMC7739139

[29] Howard DM Adams MJ Shirali M Clarke TK Marioni RE Davies G. Genome-wide association study of depression phenotypes in UK biobank identifies variants in excitatory synaptic pathways. Nat Commun. (2018) 9:1470. 10.1038/s41467-018-03819-3 29662059 PMC5902628

[30] Zhang Y Chen Y Ma L. Depression and cardiovascular disease in elderly: current understanding. J Clin Neurosci. (2018) 47:1–5. 10.1016/j.jocn.2017.09.022 29066229

[31] Yao SS Cao GY Han L Huang ZT Chen ZS Su HX. Associations between somatic multimorbidity patterns and depression in a longitudinal cohort of middle-aged and older Chinese. J Am Med Dir Assoc. (2020) 21:1282–7 e2. 10.1016/j.jamda.2019.11.028 31928934

[32] Masnoon N Shakib S Kalisch-Ellett L Caughey GE. What is polypharmacy? A systematic review of definitions. BMC Geriatr. (2017) 17:230. 10.1186/s12877-017-0621-2 29017448 PMC5635569

[33] Singh-Manoux A Fayosse A Sabia S Tabak A Shipley M Dugravot A. Clinical, socioeconomic, and behavioural factors at age 50 years and risk of cardiometabolic multimorbidity and mortality: a cohort study. PLoS Med. (2018) 15:e1002571. 10.1371/journal.pmed.1002571 29782486 PMC5962054

[34] Velten J Bieda A Scholten S Wannemuller A Margraf J. Lifestyle choices and mental health: a longitudinal survey with German and Chinese Students. BMC Public Health. (2018) 18:632. 10.1186/s12889-018-5526-2 29769115 PMC5956886

[35] Parker G Brotchie H. Gender differences in depression. Int Rev Psychiatry. (2010) 22:429–36. 10.3109/09540261.2010.492391 21047157

[36] Guertler D Moehring A Krause K Tomczyk S Freyer-Adam J Baumann S. Latent alcohol use patterns and their link to depressive symptomatology in medical care patients. Addiction. (2021) 116:1063–73. 10.1111/add.15261 32918508

[37] Hare DL Toukhsati SR Johansson P Jaarsma T. Depression and cardiovascular disease: a clinical review. Eur Heart J. (2014) 35:1365–72. 10.1093/eurheartj/eht462 24282187

[38] Kusnanto H Agustian D Hilmanto D. Biopsychosocial model of illnesses in primary care: a hermeneutic literature review. J Family Med Prim Care. (2018) 7:497–500. 10.4103/jfmpc.jfmpc_145_17 30112296 PMC6069638

[39] Fry A Littlejohns TJ Sudlow C Doherty N Adamska L Sprosen T. Comparison of sociodemographic and health-related characteristics of uk biobank participants with those of the general population. Am J Epidemiol. (2017) 186:1026–34. 10.1093/aje/kwx246 28641372 PMC5860371

[40] Strine TW Mokdad AH Dube SR Balluz LS Gonzalez O Berry JT. The association of depression and anxiety with obesity and unhealthy behaviors among community-dwelling US adults. Gen Hosp Psychiatry. (2008) 30:127–37. 10.1016/j.genhosppsych.2007.12.008 18291294

