# Supplementary material for: Association between single and multiple cardiometabolic diseases and depression: A cross-sectional study of 391,083 participants from the UK biobank
Source: Front Public Health. 2022 Aug 4;10:904876. doi: 10.3389/fpubh.2022.904876 (PMC9386503; doi:10.3389/fpubh.2022.904876)
Supplement: Supplementary file 1 [file Data_Sheet_1.docx]

**Section 1**

**Definition of depression**

This section describes the four measurements of depression symptoms used to ascertain depression disorders in the UK Biobank. (ID) refers to the corresponding UK Biobank data field ID.

1. Self-reported history of depression:

Described “depression” for medical conditions during the verbal interview at recruitment (ID: 20002) (Data-Coding 6: 1286)

1. Self-reported antidepressant using history:

Described antidepressant for medication use during the verbal interview at recruitment (ID: 20003) (Data-Coding 4: 1140879616, 1140921600, 1140879540, 1140867878, 1140916282, 1140909806, 1140867888, 1141152732, 1141180212, 1140879634, 1140867876, 1140882236, 1141190158, 1141200564, 1140867726, 1140879620, 1140867818, 1140879630, 1140879628, 1141151946, 1140867948, 1140867624, 1140867756, 1140867884, 1141151978, 1141152736, 1141201834, 1140867690, 1140867640, 1140867920, 1140867850, 1140879544, 1141200570, 1140867934, 1140867758, 1140867914, 1140867820, 1141151982, 1140882244, 1140879556, 1140867852, 1140867860, 1140917460, 1140867938, 1140867856, 1140867922, 1140910820, 1140882312, 1140867944, 1140867784, 1140867812, and 1140867668)

1. Three depression phenotypes defined by Smith, et al. ^1^:
   1. Probable lifetime (single) episode of major depression (ID: 20126) (Case-Coding: 5):

***EITHER***

- - 1. Ever depressed/down for a whole week (ID: 4598); ***AND***
    2. At least two weeks duration (ID: 4609); ***AND***
    3. Only one episode (ID: 4620); ***AND***
    4. Ever seen a GP (ID: 2090) ***OR*** a psychiatrist (ID: 2100) for nerves, anxiety, depression

***OR***

1. Ever anhedonic (unenthusiasm/uninterest) for a whole week (ID: 4631); ***AND***
2. At least two weeks duration (ID: 5375); ***AND***
3. Only one episode (ID: 5386); ***AND***
4. Ever seen a GP (ID: 2090) ***OR*** a psychiatrist (ID: 2100) for nerves, anxiety, depression
   1. Probable recurrent major depressive disorder (moderate) (ID: 20126) (Case-Coding: 4):

***EITHER***

- - 1. Ever depressed/down for a whole week (ID: 4598); ***AND***
    2. At least two weeks duration (ID: 4609); ***AND***
    3. At least two episodes (ID: 4620); ***AND***
    4. Ever seen a GP (ID: 2090) (but not a psychiatrist) for nerves, anxiety, depression

***OR***

1. Ever anhedonic (unenthusiasm/uninterest) for a whole week (ID: 4631); ***AND***
2. At least two weeks duration (ID: 5375); ***AND***
3. At least two episodes (ID: 5386); ***AND***
4. Ever seen a GP (ID: 2090) (but not a psychiatrist) for nerves, anxiety, depression
   1. Probable recurrent major depressive disorder (severe) (ID: 20126) (Case-Coding: 3):

***EITHER***

- - 1. Ever depressed/down for a whole week (ID: 4598); ***AND***
    2. At least two weeks duration (ID: 4609); ***AND***
    3. At least two episodes (ID: 4620); ***AND***
    4. Ever seen a psychiatrist (ID: 2100) for nerves, anxiety, depression

***OR***

1. Ever anhedonic (unenthusiasm/uninterest) for a whole week (ID: 4631); ***AND***
2. At least two weeks duration (ID: 5375); ***AND***
3. At least two episodes (ID: 5386); ***AND***
4. Ever seen a psychiatrist (ID: 2100) for nerves, anxiety, depression
5. Diagnosis of hospital inpatient coded by the International Classification of Disease version 10 (ICD-10):

Had recordings of hospital inpatient diagnoses for depression before the date at recruitment (ID: 41270) (Data-Coding 19: F32, F320, F321, F322, F323, F328, F329, F33, F330, F331, F332, F333, F334, F338, and F339)

**Section 2**

**Definition of psychosis phenotypes**

This section describes the four measurements of psychosis phenotypes derived from four different resources in the UK Biobank. (ID) refers to the corresponding UK Biobank data field ID.

1. Self-reported history of psychosis:

Described “schizophrenia” or “mania/bipolar disorder/manic depression” for medical conditions during the verbal interview at recruitment (ID: 20002) (Data-Coding 6: 1289 and 1291)

1. Self-reported antipsychotic medication using history:

Described antipsychotic medication for medication use during the verbal interview at recruitment (ID: 20003) (Data-Coding 4: 1140868170, 1140928916, 1141152848, 1140867444, 1140879658, 1140868120, 1141153490, 1140867304, 1141152860, 1140867168, 1141195974, 1140867244, 1140867152, 1140909800, 1140867420, 1140879746, 1141177762, 1140867456, 1140867952, 1140867150, 1141167976, 1140882100, 1140867342, 1140863416, 1141202024, 1140882098, 1140867184, 1140867092, 1140882320, 1140910358, 1140867208, 1140909802, 1140867134, 1140867306, 1140867210, 1140867398, 1140867078, 1140867218, 1141201792, 1141200458, 1140867136, 1140879750, 1140867180, 1140867546, 1140928260, and 1140927956)

1. Bipolar phenotypes defined by Smith, et al. ^1^:
   1. Bipolar Type I (Mania) (ID: 20126) (Case-Coding: 1):
      1. Ever manic/hyper 2 days (ID: 4642) ***OR*** Ever irritable/argumentative for 2 days (ID: 4653); ***AND***
      2. At least 3 from 6156.01 (more active), 6156.02 (more talkative), 6156.03 (needed less sleep), and 6156.04 (more creative/more ideas); ***AND***
      3. Duration of a week or more (ID: 5663); ***AND***
      4. Needed treatment or caused problems at work (ID:5674)
   2. Bipolar Type II (Hypomania) (ID: 20126) (Case-Coding: 1):
      1. Ever manic/hyper 2 days (ID: 4642) ***OR*** Ever irritable/argumentative for 2 days (ID: 4653); ***AND***
      2. At least 3 from 6156.01 (more active), 6156.02 (more talkative), 6156.03 (needed less sleep), and 6156.04 (more creative/more ideas); ***AND***
      3. Duration of a week or more (ID: 5663)
2. Diagnosis of hospital inpatient coded by the International Classification of Disease version 10 (ICD-10):

Had recordings of hospital inpatient diagnoses for psychosis or other mood disorders before the date at recruitment (ID: 41270) (Data-Coding 19: F20, F200, F201, F202, F203, F204, F205, F206, F208, F209, F21, F22, F220, F228, F229, F23, F230, F231, F232, F233, F238, F239, F24, F25, F250, F251, F252, F258, F259, F28, F29, F30, F300, F301, F302, F308, F309, F31, F310, F311, F312, F313, F314, F315, F316, F317, F318, F319, F34, F340, F341, F348, F349, F38, F380, F381, F388, and F39)

**References**

1. Smith DJ, Nicholl BI, Cullen B, Martin D, Ul-Haq Z, Evans J, et al. Prevalence and characteristics of probable major depression and bipolar disorder within UK biobank: cross-sectional study of 172,751 participants. *PLoS One*. 2013; **8**(11): e75362.

Supplementary Table 1 Definitions of cardiometabolic diseases

| Cardiometabolic diseases | Self-reported history | | | ICD-9 | ICD-10 | OPCS-4 |
| --- | --- | --- | --- | --- | --- | --- |
|  | Disease history | Medication | Operation |  |  |  |
| Diabetes | 2443(1), 2976, 20002(1220, 1222, 1223), 20008, 20009 | 6153(3), 6177(3) |  | 41271 (250, 3572, 3620), 41281 | 41270 (E10-E14, G590, G632, H280, H360, M142, N083), 41280, 130714, 130712, 130710, 130708, 130706, 130715, 130713, 130711, 130709, 130707 |  |
| Hypertension | 2966, 6150(4), 20002(1065, 1072), 20008, 20009 | 6153(2), 6177(2) |  | 41271 (401-405), 41281 | 41270 (I10-I13, I15, O10), 41280, 131292, 131290, 131288, 131286, 131294, 132180, 131295, 131293, 131291, 131289, 131287, 132181 |  |
| CAD | 6150(1, 2), 3894, 3627, 20002(1074, 1075), 20008, 20009 |  | 20004(1070, 1095, 1523), 20010, 20011 | 41271 (410-414), 41281 | 41270 (I20-I25, Z951, Z955), 41280, 131296, 131298, 131300, 131302, 131304, 131306, 131307, 131305, 131303, 131301, 131299, 131297 | 41272 (K40-K46, K49, K50, K75), 41282 |
| Stroke | 6150(3), 4056, 20002(1081, 1491, 1583, 1086), 20008, 20009 |  |  | 41271 (3361, 36231, 36232, 430, 431, 4329, 43301, 43311, 43321, 43331, 43381, 43391, 434, 436), 41281 | 41270 (I60, I61, I629, I63, I64, I678, I690, I693, G951, H341, H342, S066), 41280, 131378, 131376, 131374, 131372, 131370, 131368, 131366, 131364, 131362, 131360, 131180, 131379, 131377, 131375, 131373, 131371, 131369, 131367, 131365, 131363, 131361, 131181 | 41272 (A052-A054, L351, L353, L343), 41282 |

The filed IDs of the self-reported information and medical records in the UK Biobank, which were used to define the prevalence and/or incidence of the four cardiometabolic diseases in individuals are presented.

CAD, Coronary artery disease; ICD, International Classification of Diseases; OPCS, the Office of Population Censuses and Surveys Classification of Interventions and Procedures.

Supplementary Table 2 The calculation of healthy diet score

| Healthy food items | Intake | Notes |
| --- | --- | --- |
| Total fruit and vegetable | >4.5 pieces or servings per day | 3 tablespoons of vegetables were considered 1 serving |
| Total fish | >2 times per week |  |
| Processed and red meat | ≤2 times of processed meat per week | Conditions for both processed and red meat intake need to be fulfilled |
|  | ≤5times of red meat per week |  |

The healthy diet score was adapted from the American Heart Association Guidelines, involving food items including fruit and vegetable, fish, processed and red meat intake. If ≥2 healthy food items are fulfilled, healthy diet score is 1 (more advisable), otherwise healthy diet score is 0 (less advisable). The information on the intake of food items could be derived from UK Biobank directly.

Supplementary Table 3 Population characteristics according to status of CMDs *

| Characteristics | No CMDs | CAD | DM | HT | ST | HT+CAD | HT+DM | HT+ST | HT+CAD+DM | Others |
| --- | --- | --- | --- | --- | --- | --- | --- | --- | --- | --- |
| Participants, n (%) | 273803 (70.0%) | 3986 (1.0%) | 5224 (1.3%) | 79853 (20.4%) | 2191 (0.6%) | 9338 (2.4%) | 9446 (2.4%) | 2742 (0.7%) | 2366 (0.6%) | 2134 (0.5%) |
| Age (years), mean (SD) | 54.84 (8.03) | 60.70 (6.86) | 56.66 (8.04) | 58.74 (7.32) | 58.21 (7.58) | 62.02 (6.09) | 59.66 (6.96) | 61.19 (6.63) | 62.16 (5.94) | 61.87 (6.36) |
| Male, n (%) | 114768 (41.9) | 2421 (60.7) | 2753 (52.7) | 38649 (48.4) | 1038 (47.4) | 6720 (72.0) | 5701 (60.4) | 1592 (58.1) | 1785 (75.4) | 1466 (68.7) |
| White ethnicity, n (%) | 261198 (95.4) | 3793 (95.2) | 4567 (87.4) | 75728 (94.8) | 2124 (96.9) | 8883 (95.1) | 8317 (88.0) | 2639 (96.2) | 2067 (87.4) | 1936 (90.7) |
| Townsend Deprivation Index, mean (SD) | -1.63 (2.89) | -1.36 (3.07) | -1.05 (3.21) | -1.56 (2.96) | -1.38 (3.06) | -1.39 (3.07) | -0.91 (3.22) | -1.29 (3.09) | -0.62 (3.39) | -0.59 (3.40) |
| College and higher degree #, n (%) | 161285 (58.9) | 2240 (56.2) | 2929 (56.1) | 44088 (55.2) | 1193 (54.5) | 4885 (52.3) | 5105 (54.0) | 1443 (52.6) | 1160 (49.0) | 1079 (50.6) |
| Healthy diet, n (%) | 153836 (56.2) | 2323 (58.3) | 2920 (55.9) | 46140 (57.8) | 1249 (57.0) | 5511 (59.0) | 5295 (56.1) | 1560 (56.9) | 1316 (55.6) | 1222 (57.3) |
| Current smoking, n (%) | 26193 (9.6) | 477 (12.0) | 573 (11.0) | 6474 (8.1) | 295 (13.5) | 883 (9.5) | 813 (8.6) | 315 (11.5) | 240 (10.1) | 274 (12.8) |
| Alcohol consumption frequency (times/week) ≥3, n (%) | 127628 (46.6) | 1817 (45.6) | 1754 (33.6) | 38626 (48.4) | 922 (42.1) | 4496 (48.1) | 3247 (34.4) | 1213 (44.2) | 778 (32.9) | 747 (35.0) |
| Body mass index (kg/m^2^), mean (SD) | 26.38 (4.26) | 27.51 (4.41) | 29.11 (5.56) | 28.76 (5.00) | 27.00 (4.38) | 29.03 (4.81) | 31.98 (5.96) | 28.47 (4.80) | 31.73 (5.72) | 30.22 (5.47) |
| Depression disorder, n (%) | 38705 (14.1) | 666 (16.7) | 934 (17.9) | 13364 (16.7) | 472 (21.5) | 1610 (17.2) | 1758 (18.6) | 610 (22.2) | 530 (22.4) | 533 (25.0) |
| Antidepressant use, n (%) | 15212 (5.6) | 307 (7.7) | 468 (9.0) | 6168 (7.7) | 229 (10.5) | 797 (8.5) | 987 (10.4) | 327 (11.9) | 300 (12.7) | 303 (14.2) |

CMDs means cardiometabolic diseases; CAD means coronary artery disease; DM means diabetes mellitus; HT means hypertension; ST means stroke.

* The status of CMDs was classified exclusively to ten patterns as following: no CMDs, CAD, DM, HT, ST, HT+CAD, HT+DM, HT+ST, HT+CAD+DM, and the combination of other CMD patterns with cases <1000, i.e., others in the table.

# College and higher degree included college or university degree and other professional qualifications such as nursing and teaching.

Supplementary Table 4 The association between CMD patterns and depression §

| CMD patterns | Base model * | | |  | Partially-adjusted model # | | |  | Full-adjusted model & | | |
| --- | --- | --- | --- | --- | --- | --- | --- | --- | --- | --- | --- |
|  | OR | 95% CI | *P* |  | OR | 95% CI | *P* |  | OR | 95% CI | *P* |
| CAD | 1.47 | (1.35; 1.60) | <0.001 |  | 1.43 | (1.31; 1.56) | <0.001 |  | 1.36 | (1.25; 1.48) | <0.001 |
| Diabetes | 1.48 | (1.38; 1.59) | <0.001 |  | 1.45 | (1.35; 1.56) | <0.001 |  | 1.31 | (1.22; 1.41) | <0.001 |
| Hypertension | 1.34 | (1.31; 1.37) | <0.001 |  | 1.33 | (1.30; 1.36) | <0.001 |  | 1.24 | (1.21; 1.27) | <0.001 |
| Stroke | 1.79 | (1.61; 1.98) | <0.001 |  | 1.75 | (1.58; 1.95) | <0.001 |  | 1.68 | (1.51; 1.86) | <0.001 |
| Hypertension + CAD | 1.69 | (1.59; 1.78) | <0.001 |  | 1.64 | (1.55; 1.73) | <0.001 |  | 1.51 | (1.43; 1.60) | <0.001 |
| Hypertension + diabetes | 1.70 | (1.61; 1.79) | <0.001 |  | 1.64 | (1.55; 1.73) | <0.001 |  | 1.36 | (1.29; 1.44) | <0.001 |
| Hypertension + stroke | 2.07 | (1.89; 2.27) | <0.001 |  | 2.01 | (1.84; 2.21) | <0.001 |  | 1.87 | (1.70; 2.05) | <0.001 |
| Hypertension + diabetes + CAD | 2.46 | (2.23; 2.72) | <0.001 |  | 2.32 | (2.10; 2.57) | <0.001 |  | 1.95 | (1.76; 2.16) | <0.001 |

CMD means cardiometabolic disease; CAD means coronary artery disease; OR means odds ratio; 95% CI means 95% confidence interval.

§ Reference group: participants free of CMDs.

* Base model: Adjusted for age, sex, and ethnicity.

# Partially-adjusted model: Further adjusted for Townsend deprivation score, and education degree.

& Full-adjusted model: Further adjusted for healthy diet, current smoking status, alcohol consumption, and body mass index

Supplement Table 5 The association between CMD patterns and probable lifetime depression§

| Probable lifetime depression | CMD patterns | Base model * | | |  | Partially-adjusted model # | | |  | Full-adjusted model & | | |
| --- | --- | --- | --- | --- | --- | --- | --- | --- | --- | --- | --- | --- |
|  |  | OR | 95% CI | *P* |  | OR | 95% CI | *P* |  | OR | 95% CI | *P* |
| Single episode ¶ | CAD | 1.04 | (0.82; 1.31) | 0.763 |  | 1.03 | (0.81; 1.30) | 0.825 |  | 1.00 | (0.79; 1.27) | 0.991 |
|  | Diabetes | 1.15 | (0.95; 1.39) | 0.165 |  | 1.13 | (0.94; 1.37) | 0.196 |  | 1.07 | (0.88; 1.29) | 0.520 |
|  | Hypertension | 1.18 | (1.12; 1.25) | <0.001 |  | 1.18 | (1.12; 1.25) | <0.001 |  | 1.14 | (1.07; 1.20) | <0.001 |
|  | Stroke | 1.36 | (1.04; 1.78) | 0.027 |  | 1.35 | (1.03; 1.77) | 0.029 |  | 1.32 | (1.01; 1.73) | 0.044 |
|  | Hypertension + CAD | 1.26 | (1.08; 1.46) | 0.003 |  | 1.25 | (1.07; 1.45) | 0.004 |  | 1.19 | (1.02; 1.38) | 0.025 |
|  | Hypertension + diabetes | 1.11 | (0.95; 1.29) | 0.197 |  | 1.09 | (0.94; 1.27) | 0.263 |  | 0.97 | (0.83; 1.13) | 0.692 |
|  | Hypertension + stroke | 1.33 | (1.03; 1.72) | 0.029 |  | 1.32 | (1.02; 1.71) | 0.033 |  | 1.26 | (0.97; 1.63) | 0.078 |
|  | Hypertension + diabetes + CAD | 1.55 | (1.18; 2.04) | 0.002 |  | 1.52 | (1.16; 2.00) | 0.003 |  | 1.36 | (1.03; 1.79) | 0.030 |
| Recurrent moderate ※ | CAD | 1.26 | (1.05; 1.51) | 0.012 |  | 1.23 | (1.03; 1.48) | 0.023 |  | 1.19 | (0.99; 1.42) | 0.061 |
|  | Diabetes | 1.24 | (1.07; 1.44) | 0.005 |  | 1.22 | (1.05; 1.41) | 0.010 |  | 1.12 | (0.96; 1.30) | 0.135 |
|  | Hypertension | 1.22 | (1.17; 1.28) | <0.001 |  | 1.22 | (1.16; 1.27) | <0.001 |  | 1.14 | (1.09; 1.19) | <0.001 |
|  | Stroke | 1.39 | (1.12; 1.73) | 0.003 |  | 1.38 | (1.11; 1.71) | 0.004 |  | 1.34 | (1.08; 1.67) | 0.008 |
|  | Hypertension + CAD | 1.43 | (1.26; 1.61) | <0.001 |  | 1.40 | (1.24; 1.58) | <1e-04 |  | 1.31 | (1.16; 1.48) | <0.001 |
|  | Hypertension + diabetes | 1.32 | (1.17; 1.48) | <0.001 |  | 1.28 | (1.14; 1.44) | <1e-04 |  | 1.11 | (0.98; 1.25) | 0.102 |
|  | Hypertension + stroke | 1.41 | (1.15; 1.74) | 0.001 |  | 1.39 | (1.13; 1.71) | 0.002 |  | 1.30 | (1.06; 1.60) | 0.013 |
|  | Hypertension + diabetes + CAD | 1.84 | (1.48; 2.28) | <0.001 |  | 1.77 | (1.42; 2.19) | <1e-04 |  | 1.53 | (1.23; 1.90) | <0.001 |
| Recurrent moderate ※ | CAD | 1.77 | (1.44; 2.17) | <0.001 |  | 1.68 | (1.37; 2.06) | <0.001 |  | 1.62 | (1.32; 1.98) | <0.001 |
|  | Diabetes | 1.53 | (1.28; 1.83) | <0.001 |  | 1.47 | (1.22; 1.76) | <0.001 |  | 1.37 | (1.14; 1.65) | 0.001 |
|  | Hypertension | 1.28 | (1.20; 1.36) | <0.001 |  | 1.26 | (1.18; 1.33) | <0.001 |  | 1.21 | (1.14; 1.29) | <0.001 |
|  | Stroke | 2.02 | (1.58; 2.59) | <0.001 |  | 1.95 | (1.53; 2.51) | <0.001 |  | 1.86 | (1.45; 2.39) | <0.001 |
|  | Hypertension + CAD | 1.49 | (1.28; 1.74) | <0.001 |  | 1.43 | (1.23; 1.66) | <0.001 |  | 1.36 | (1.17; 1.59) | <0.001 |
|  | Hypertension + diabetes | 1.38 | (1.19; 1.60) | <0.001 |  | 1.29 | (1.11; 1.49) | 0.001 |  | 1.17 | (1.00; 1.36) | 0.045 |
|  | Hypertension + stroke | 1.74 | (1.36; 2.22) | <0.001 |  | 1.66 | (1.29; 2.12) | <0.001 |  | 1.57 | (1.23; 2.01) | <0.001 |
|  | Hypertension + diabetes + CAD | 2.32 | (1.82; 2.94) | <0.001 |  | 2.09 | (1.65; 2.66) | <0.001 |  | 1.89 | (1.48; 2.40) | <0.001 |

CMD means cardiometabolic disease; CAD means coronary artery disease; OR means odds ratio; 95% CI means 95% confidence interval.

§ Reference group: participants free of CMDs.

* Base model: Adjusted for age, sex, and ethnicity.

# Partially-adjusted model: Further adjusted for Townsend deprivation score, and education degree.

& Full-adjusted model: Further adjusted for healthy diet, current smoking status, alcohol consumption, and body mass index.

¶ Single episode: Probable lifetime (single) episode of major depression.

※ Recurrent moderate: Probable recurrent major depressive disorder (moderate).

$ Recurrent severe: Probable recurrent major depressive disorder (severe).

Supplementary Table 6 Sensitivity analysis 1: additional adjusted for physical activity in the full-adjusted model §

| Stratifications |  | Base model * | | |  | Partially-adjusted model # | | |  | Full-adjusted model & | | |
| --- | --- | --- | --- | --- | --- | --- | --- | --- | --- | --- | --- | --- |
|  |  | OR | 95% CI | *P* |  | OR | 95% CI | *P* |  | OR | 95% CI | *P* |
| CMD patterns | CAD | 1.45 | (1.31; 1.61) | <0.001 |  | 1.42 | (1.28; 1.58) | <0.001 |  | 1.36 | (1.22; 1.51) | <0.001 |
|  | Diabetes | 1.45 | (1.33; 1.58) | <0.001 |  | 1.42 | (1.30; 1.55) | <0.001 |  | 1.29 | (1.18; 1.41) | <0.001 |
|  | Hypertension | 1.31 | (1.27; 1.34) | <0.001 |  | 1.29 | (1.26; 1.33) | <0.001 |  | 1.21 | (1.18; 1.25) | <0.001 |
|  | Stroke | 1.78 | (1.57; 2.02) | <0.001 |  | 1.75 | (1.54; 1.99) | <0.001 |  | 1.68 | (1.48; 1.91) | <0.001 |
|  | Hypertension +CAD | 1.62 | (1.51; 1.74) | <0.001 |  | 1.58 | (1.47; 1.70) | <0.001 |  | 1.47 | (1.37; 1.58) | <0.001 |
|  | Hypertension + diabetes | 1.63 | (1.53; 1.75) | <0.001 |  | 1.58 | (1.47; 1.69) | <0.001 |  | 1.31 | (1.23; 1.41) | <0.001 |
|  | Hypertension + stroke | 1.97 | (1.76; 2.21) | <0.001 |  | 1.92 | (1.71; 2.15) | <0.001 |  | 1.79 | (1.59; 2.00) | <0.001 |
|  | Hypertension + diabetes + CAD | 2.43 | (2.15; 2.75) | <0.001 |  | 2.31 | (2.04; 2.61) | <0.001 |  | 1.94 | (1.71; 2.20) | <0.001 |
| CMD numbers | 1 | 1.33 | (1.30; 1.37) | <0.001 |  | 1.32 | (1.29; 1.35) | <0.001 |  | 1.23 | (1.20; 1.27) | <0.001 |
|  | 2 | 1.69 | (1.61; 1.77) | <0.001 |  | 1.64 | (1.57; 1.72) | <0.001 |  | 1.45 | (1.38; 1.52) | <0.001 |
|  | ≥3 | 2.59 | (2.36; 2.85) | <0.001 |  | 2.45 | (2.23; 2.69) | <0.001 |  | 2.09 | (1.89; 2.30) | <0.001 |
|  | Additive dose effect | 1.33 | (1.31; 1.36) | <0.001 |  | 1.31 | (1.29; 1.34) | <0.001 |  | 1.23 | (1.21; 1.26) | <0.001 |

CMD means cardiometabolic disease; CAD means coronary artery disease; OR means odds ratio; 95% CI means 95% confidence interval.

§ Reference group: participants free of CMDs (except for the additive dose effects of CMD number).

* Base model: Adjusted for age, sex, and ethnicity.

# Partially-adjusted model: Further adjusted for Townsend deprivation score, and education degree.

& Full-adjusted model: Further adjusted for physical activity, healthy diet, current smoking status, alcohol consumption, and body mass index.

Supplementary Table 7 Sensitivity analysis 2: widening the definition of depression status by adding a measurement of help-seeking§

| Stratifications |  | Base model * | | |  | Partially-adjusted model # | | |  | Full-adjusted model & | | |
| --- | --- | --- | --- | --- | --- | --- | --- | --- | --- | --- | --- | --- |
|  |  | OR | 95% CI | *P* |  | OR | 95% CI | *P* |  | OR | 95% CI | *P* |
| CMD patterns | CAD | 1.53 | (1.42; 1.65) | <0.001 |  | 1.48 | (1.37; 1.60) | <0.001 |  | 1.42 | (1.32; 1.53) | <0.001 |
|  | Diabetes | 1.42 | (1.33; 1.52) | <0.001 |  | 1.38 | (1.29; 1.48) | <0.001 |  | 1.28 | (1.20; 1.37) | <0.001 |
|  | Hypertension | 1.31 | (1.29; 1.34) | <0.001 |  | 1.30 | (1.27; 1.32) | <0.001 |  | 1.24 | (1.21; 1.26) | <0.001 |
|  | Stroke | 1.72 | (1.56; 1.89) | <0.001 |  | 1.68 | (1.52; 1.84) | <0.001 |  | 1.62 | (1.47; 1.78) | <0.001 |
|  | Hypertension +CAD | 1.64 | (1.55; 1.72) | <0.001 |  | 1.58 | (1.50; 1.66) | <0.001 |  | 1.49 | (1.42; 1.57) | <0.001 |
|  | Hypertension + diabetes | 1.60 | (1.52; 1.68) | <0.001 |  | 1.53 | (1.45; 1.60) | <0.001 |  | 1.34 | (1.27; 1.41) | <0.001 |
|  | Hypertension + stroke | 1.90 | (1.75; 2.07) | <0.001 |  | 1.84 | (1.69; 2.00) | <0.001 |  | 1.73 | (1.59; 1.89) | <0.001 |
|  | Hypertension + diabetes + CAD | 2.29 | (2.09; 2.50) | <0.001 |  | 2.13 | (1.94; 2.34) | <0.001 |  | 1.88 | (1.71; 2.06) | <0.001 |
| CMD number | 1 | 1.34 | (1.31; 1.36) | <0.001 |  | 1.32 | (1.29; 1.34) | <0.001 |  | 1.26 | (1.23; 1.28) | <0.001 |
|  | 2 | 1.67 | (1.62; 1.73) | <0.001 |  | 1.61 | (1.55; 1.66) | <0.001 |  | 1.47 | (1.42; 1.52) | <0.001 |
|  | ≥3 | 2.47 | (2.31; 2.65) | <0.001 |  | 2.30 | (2.14; 2.47) | <0.001 |  | 2.04 | (1.90; 2.19) | <0.001 |
|  | Additive dose effect | 1.33 | (1.31; 1.35) | <0.001 |  | 1.30 | (1.29; 1.32) | <0.001 |  | 1.24 | (1.23; 1.26) | <0.001 |

The definition of depression was broadened by adding a measurement of help-seeking. Participants who had ever seen a psychiatrist for nerves, anxiety, tension, or depression were thought to fulfill this criterion. CMD means cardiometabolic diseases; CAD means coronary artery disease; OR means odds ratio; 95% CI means 95% confidence interval.

§ Reference group: participants free of CMDs (except for the additive dose effects of CMD number).

* Base model: Adjusted for age, sex, and ethnicity.

# Partially-adjusted model: Further adjusted for Townsend deprivation score, and education degree.

& Full-adjusted model: Further adjusted for healthy diet, current smoking status, alcohol consumption, and body mass index.

Supplementary Table 8 Sensitivity analysis 3: modifying the definition of depression status by adding a measurement of PHQ-2 §

| Stratifications |  | Base model * | | |  | Partially-adjusted model # | | |  | Full-adjusted model & | | |
| --- | --- | --- | --- | --- | --- | --- | --- | --- | --- | --- | --- | --- |
|  |  | OR | 95% CI | *P* |  | OR | 95% CI | *P* |  | OR | 95% CI | *P* |
| CMD patterns | CAD | 1.62 | (1.52; 1.73) | <0.001 |  | 1.54 | (1.45; 1.65) | <0.001 |  | 1.47 | (1.38; 1.57) | <0.001 |
|  | Diabetes | 1.57 | (1.48; 1.66) | <0.001 |  | 1.51 | (1.43; 1.60) | <0.001 |  | 1.37 | (1.29; 1.45) | <0.001 |
|  | Hypertension | 1.34 | (1.31; 1.36) | <0.001 |  | 1.31 | (1.29; 1.34) | <0.001 |  | 1.23 | (1.21; 1.25) | <0.001 |
|  | Stroke | 1.84 | (1.69; 2.01) | <0.001 |  | 1.79 | (1.64; 1.94) | <0.001 |  | 1.70 | (1.56; 1.86) | <0.001 |
|  | Hypertension +CAD | 1.81 | (1.73; 1.89) | <0.001 |  | 1.71 | (1.64; 1.78) | <0.001 |  | 1.58 | (1.51; 1.65) | <0.001 |
|  | Hypertension + diabetes | 1.75 | (1.67; 1.82) | <0.001 |  | 1.65 | (1.58; 1.72) | <0.001 |  | 1.39 | (1.33; 1.46) | <0.001 |
|  | Hypertension + stroke | 2.08 | (1.93; 2.24) | <0.001 |  | 1.98 | (1.84; 2.13) | <0.001 |  | 1.83 | (1.70; 1.97) | <0.001 |
|  | Hypertension + diabetes + CAD | 2.70 | (2.51; 2.90) | <0.001 |  | 2.48 | (2.30; 2.66) | <0.001 |  | 2.09 | (1.94; 2.25) | <0.001 |
| CMD number | 1 | 1.38 | (1.35; 1.40) | <0.001 |  | 1.35 | (1.33; 1.37) | <0.001 |  | 1.26 | (1.24; 1.29) | <0.001 |
|  | 2 | 1.84 | (1.79; 1.89) | <0.001 |  | 1.74 | (1.69; 1.79) | <0.001 |  | 1.54 | (1.50; 1.59) | <0.001 |
|  | ≥3 | 2.84 | (2.69; 3.01) | <0.001 |  | 2.59 | (2.45; 2.74) | <0.001 |  | 2.21 | (2.09; 2.34) | <0.001 |
|  | Additive dose effect | 1.39 | (1.38; 1.41) | <0.001 |  | 1.35 | (1.34; 1.37) | <0.001 |  | 1.27 | (1.26; 1.29) | <0.001 |

The definition of depression was broadened by adding a measurement of PHQ-2. Based on PHQ-2, participants were considered with probable current depression or not according to their self-reported frequency of depressed mood and unenthusiasm/disinterest during the last 2 weeks at recruitment. PHQ-2 means two-item Patient Health Questionnaire; CMD means cardiometabolic diseases; CAD means coronary artery disease; OR means odds ratio; 95% CI means 95% confidence interval.

§ Reference group: participants free of CMDs (except for the additive dose effects of CMD number).

* Base model: Adjusted for age, sex, and ethnicity.

# Partially-adjusted model: Further adjusted for Townsend deprivation score, and education degree.

& Full-adjusted model: Further adjusted for healthy diet, current smoking status, alcohol consumption, and body mass index.

Supplementary Table 9 Sensitivity analysis 4: modifying the assessment of education degree §

| Stratifications |  | Base model * | | |  | Partially-adjusted model # | | |  | Full-adjusted model & | | |
| --- | --- | --- | --- | --- | --- | --- | --- | --- | --- | --- | --- | --- |
|  |  | OR | 95% CI | *P* |  | OR | 95% CI | *P* |  | OR | 95% CI | *P* |
| CMD patterns | CAD | 1.47 | (1.35; 1.60) | <0.001 |  | 1.43 | (1.31; 1.56) | <0.001 |  | 1.36 | (1.25; 1.48) | <0.001 |
|  | Diabetes | 1.48 | (1.38; 1.59) | <0.001 |  | 1.45 | (1.35; 1.56) | <0.001 |  | 1.31 | (1.22; 1.41) | <0.001 |
|  | Hypertension | 1.34 | (1.31; 1.37) | <0.001 |  | 1.33 | (1.30; 1.36) | <0.001 |  | 1.24 | (1.21; 1.27) | <0.001 |
|  | Stroke | 1.79 | (1.61; 1.98) | <0.001 |  | 1.75 | (1.58; 1.95) | <0.001 |  | 1.68 | (1.51; 1.87) | <0.001 |
|  | Hypertension + CAD | 1.69 | (1.59; 1.78) | <0.001 |  | 1.64 | (1.55; 1.73) | <0.001 |  | 1.51 | (1.43; 1.60) | <0.001 |
|  | Hypertension + diabetes | 1.70 | (1.61; 1.79) | <0.001 |  | 1.64 | (1.55; 1.73) | <0.001 |  | 1.36 | (1.29; 1.44) | <0.001 |
|  | Hypertension + stroke | 2.07 | (1.89; 2.27) | <0.001 |  | 2.01 | (1.84; 2.21) | <0.001 |  | 1.87 | (1.70; 2.05) | <0.001 |
|  | Hypertension + diabetes + CAD | 2.46 | (2.23; 2.72) | <0.001 |  | 2.32 | (2.10; 2.57) | <0.001 |  | 1.95 | (1.76; 2.16) | <0.001 |
| CMD number | 1 | 1.37 | (1.34; 1.39) | <0.001 |  | 1.35 | (1.32; 1.38) | <0.001 |  | 1.26 | (1.23; 1.29) | <0.001 |
|  | 2 | 1.76 | (1.70; 1.83) | <0.001 |  | 1.70 | (1.64; 1.77) | <0.001 |  | 1.50 | (1.44; 1.56) | <0.001 |
|  | ≥3 | 2.66 | (2.46; 2.87) | <0.001 |  | 2.50 | (2.32; 2.70) | <0.001 |  | 2.13 | (1.97; 2.30) | <0.001 |
|  | Additive dose effect | 1.36 | (1.34; 1.38) | <0.001 |  | 1.34 | (1.32; 1.36) | <0.001 |  | 1.26 | (1.24; 1.27) | <0.001 |

The education degree was assessed with a more fine-grained ordinal variable: college/university/other professional qualification; A-levels/AS-levels; O-levels/GCSEs; CSEs; NVQ/HND/HNC. CMD means cardiometabolic disease; CAD means coronary artery disease; OR means odds ratio; 95% CI means 95% confidence interval.

§ Reference group: participants free of CMDs (except for the additive dose effects of CMD number).

* Base model: Adjusted for age, sex, and ethnicity.

# Partially-adjusted model: Further adjusted for Townsend deprivation score, and education degree.

& Full-adjusted model: Further adjusted for healthy diet, current smoking status, alcohol consumption, and body mass index.
